# An endogenous mCherry-tagged COSA-1 as a crossover investigation tool in *Caenorhabditis elegans*

**DOI:** 10.17912/micropub.biology.000627

**Published:** 2022-08-16

**Authors:** Chiemekam Samuel Ezechukwu, Arome Solomon Odiba, Guiyan Liao, Wenxia Fang, Bin Wang

**Affiliations:** 1 State Key Laboratory of Non-food Biomass and Enzyme Technology, Guangxi Academy of Sciences, Nanning 530007, China; 2 Department of Zoology and Environmental Biology, University of Nigeria, Nsukka 410001, Nigeria

## Abstract

The *C. elegans* *cosa-1* gene encodes the crossover site-associated-1 (COSA-1) protein, a cyclin-related protein that functions in promoting crossovers (COs) during meiosis. Previous studies regarding CO dynamics in live *C. elegans* have mostly relied on the green fluorescent protein-tagged *cosa-1* transgenic strain, which was generated by the microparticle bombardment method. Here, we insert the red fluorescence protein mCherry at the C-terminal of the *cosa-1* gene to establish *cosa-1::mCherry* transgenic worm by the CRISPR/Cas9 technique. The COSA-1::mCherry was observed to appear from the early pachytene, and disappear in the diplotene zone of the germline, with 6 COSA-1:: mCherry foci in the late pachytene, which colocalized with GFP::COSA-1 from AV630 strain. Furthermore, the transgenic strain harboring a *cosa-1::mCherry* fusion shows no defect in the brood size, progeny viability and male frequency, which provides a useful tool for the meiotic analysis in *C. elegans* .

**Figure 1. Cytological and phenotypic characterization of cosa-1::mCherry(wsh7) .**
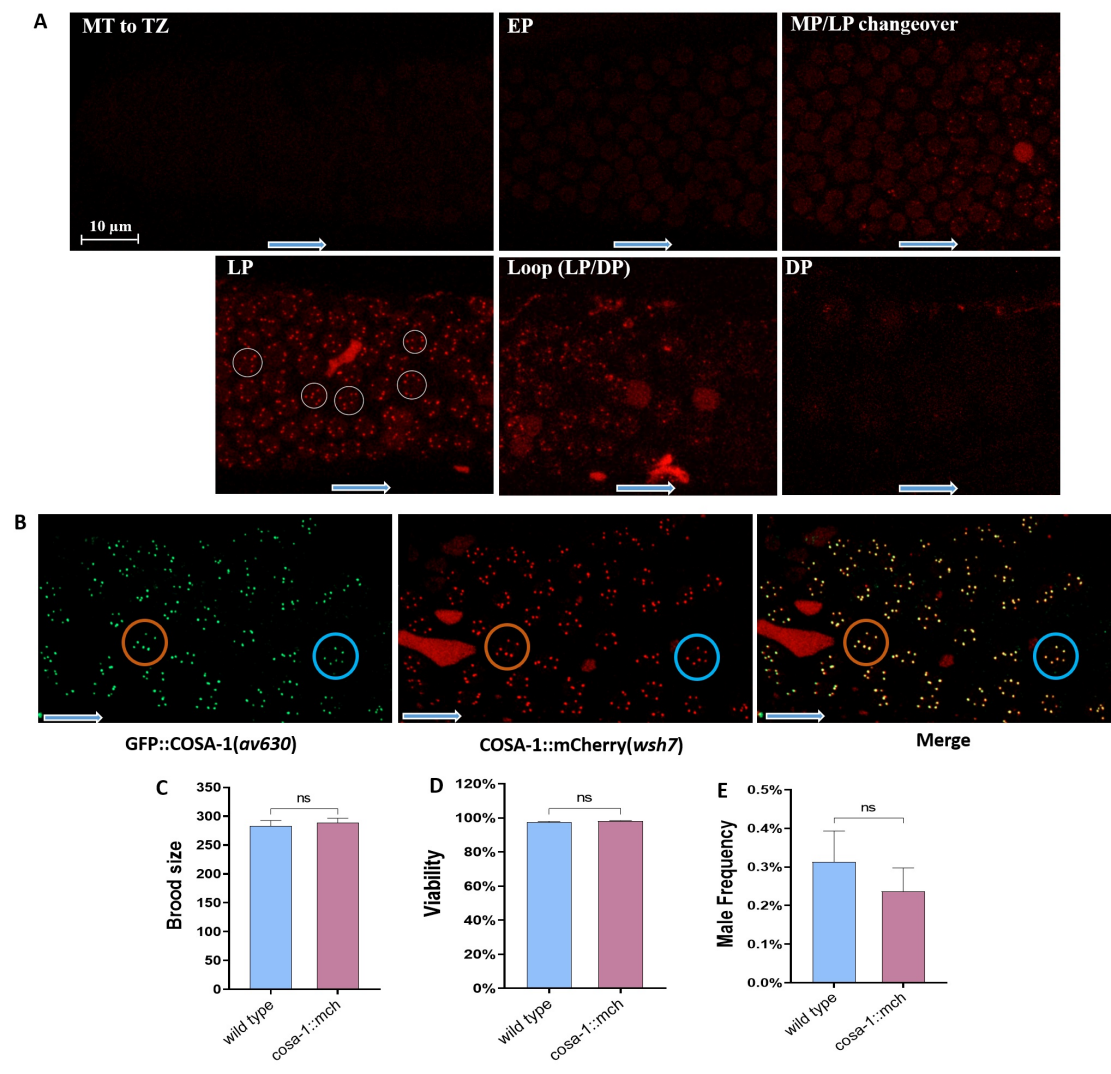
(A) Fluorescence images showing the localization of the COSA-1::mCherry foci in the different zones of the germline. White rings indicate examples of 6 COSA-1::mCherry foci per nuclei in the late pachytene (LP) of adult worms, blue arrows define germline distal to proximal direction (MZ - mitotic zone, TZ - transition zone, EP - early pachytene, MP - mid pachytene, LP - late pachytene, DP -diplotene). (B) Colocalization of GFP::COSA-1 and COSA-1::mCherry. (C - E) Analysis of brood size (C), progeny viability (D) and male frequency (E) in the *cosa-1::mCherry(wsh7)* transgenic strain compared with the wild type (ns - not significant; Independent samples t-test, n= 26).

## Description

The *Caenorhabditis elegans* *cosa-1* encodes the crossover site-associated-1 (COSA-1) protein, a cyclin-related protein, which is widely conserved across metazoans and functions in the conversion of the designated double strand breaks (DSBs) to crossovers (COs) during meiosis (Yokoo et al., 2012). COSA-1 is normally localized to presumptive CO sites during meiotic recombination, and there is only 1 CO event per homologous chromosome pair in *C. elegans* , which is consistent with 6 GFP::COSA-1 foci observed in late pachytene germ cells corresponding to the six chromosome pairs (Yokoo et al. 2012; Li et al., 2018). This green fluorescent protein (GFP)-tagged *cosa-1* strain AV630 ( *meIs8 [pie-1p::GFP::cosa-1 + unc-119(+)]* II), was generated by the microparticle bombardment method, which is widely used to analyze the meiotic CO dynamics and interference (Yokoo *et al* ., 2012; Li et al., 2018; Girard et al., 2021; Gordon et al., 2021).

To check the expression pattern of endogenous *cosa-1* in *C. elegans* , we tagged the 3’ end of endogenous *cosa-1* coding region with the red fluorescent protein mCherry by the CRISPR/Cas9 technique to generate the *cosa-1::mCherry* *knock-in* strain. The *cosa-1::mCherry knock-in* showed mCherry expression in the nucleus from the early pachytene to the diplotene zone, with 6 COSA-1::mCherry foci in the late pachytene (Figure 1A), which colocalized with GFP::COSA-1 foci from AV630 strain (Figure 1B, Yokoo et al., 2012). However, we observed that COSA-1::mCherry foci disappeared earlier (during diplotene) than GFP::COSA-1 (Figure 1A), which might be a consequence of the strategy in strain construction (Yokoo et al., 2012). While the *GFP::cosa-1* was driven by the *pie-1* promoter and integrated in the other locus on the chromosome by microparticle bombardment, the *cosa-1::mCherry* that tagged the fluorescence to the endogenous locus of the *cosa-1* gene was driven by its native promoter.

Moreover, we compared the *cosa-1::mCherry(wsh7)* strain to wild-type and observed no significant difference in brood size (Figure 1C, P values = 0.63861), progeny viability (Figure 1D, P values = 0.1998) and male frequency (Figure 1E, P values = 0.4491), suggesting there is no functional defect in the *cosa-1::mCherry(wsh7)* . Therefore, *cosa-1::mCherry(wsh7)* should be a useful tool strain to the CO analysis in *C. elegans* meiosis.

## Methods

The *cosa-1::mCherry* was generated using the CRISPR/Cas9 method. The 5’ TGTCAGAGATGGTAGTTACGAGG 3’ sgRNA sequence was used to generate the U6 promoter::sgRNA templates by PCR as described (Ward 2014), which directs the cleavage to the C-terminal of *cosa-1* gene. Similarly, the *cosa-1::mCherry* template was constructed by fusion PCR as follows: ~1000bp upstream DNA sequence before the stop codon of the *cosa-1* gene ( *cosa-1::mCherry* UF: TTTGCCTCGTCCCTCGTG, *cosa-1::mCherry* UR: ACCGATCCCCCGGGCACGAGGAGGTGCTGCATTCC) was added before the start codon of the *mCherry* coding sequence (PCR from plasmid pKD233.7-3, mch-F: TGCCCGGGGGATCGGT, mch-R: TTACTTGTACAGCTCGTCCATGCC), and ~1000 bp downstream DNA sequence after the stop codon of the *cosa-1* gene ( *cosa-1::mCherry* DF: GGCATGGACGAGCTGTACAAGTAACTACCATCTCTGACAGCACCTC, *cosa-1::mCherry* DR: CCAACGGGATTTCGGAGTA) was added before the stop codon of the *mCherry* coding sequence. The constructed *cosa-1::mCherry* template was cloned to the T-vector and sequenced. The mixture of pDD162 ( *Peft-3::Cas9* , 50 ng/µL), pCFJ90 ( *Pmyo-2* :: *mCherry* , 2.5 ng/µL) and pCFJ104 ( *Pmyo-3::mCherry* , 5 ng/µL) plasmids (Dickinson et al., 2013), together with pU6-cosa-1 sgRNA (50 ng/µL) and *cosa-1::mCherry* repair templates (50 ng/µL), was microinjected in N2 young adult worms. Worms expressing the selection markers were picked out, which were further screened for successfully integrated *cosa-1::mCherry* by PCR using forward primer 5’-TGCGCGAAAAAGGTAACTGC-3’ and reverse primer 5’ ACGTGACAGGAAATTGCGAA 3’. The *cosa-1::mCherry* transgenic was backcrossed 4 times and confirmed by the sequencing. All images were captured using a Zeiss LSM800 confocal microscope with the 63X objective.

For the phenotypic characterization, L4 hermaphrodites were plated (1 worm per plate) onto NGM plates seeded with M9-diluted OP50 in the center, and maintained at 20 ^o^ C. The worms were transferred into freshly seeded NGM plates at every 12 hours interval, and this was continued until worm ceased egg laying. The total laid eggs were counted, followed by unhatched eggs and number of males counted after 24 hours and 72 hours respectively. The viability was calculated as the percentage of hatched eggs over the total number of laid eggs. Independent sample t-test was used to compare the means of the brood size (total number of eggs laid), viability and male frequency by GraphPad Prism 8.0 software.

## Reagents

**Strain:** XSW955, *cosa-1(wsh7 [cosa-1::mCherry])* III

## References

[R1] Dickinson DJ, Ward JD, Reiner DJ, Goldstein B (2013). Engineering the Caenorhabditis elegans genome using Cas9-triggered homologous recombination.. Nat Methods.

[R2] Frøkjaer-Jensen C, Davis MW, Hopkins CE, Newman BJ, Thummel JM, Olesen SP, Grunnet M, Jorgensen EM (2008). Single-copy insertion of transgenes in Caenorhabditis elegans.. Nat Genet.

[R3] Girard C, Akerib CC, Villeneuve AM (2021). Suppression of
*him-14(it44ts)*
by a transgene insertion expressing GFP::COSA-1.. MicroPubl Biol.

[R4] Gordon SG, Kursel LE, Xu K, Rog O (2021). Synaptonemal Complex dimerization regulates chromosome alignment and crossover patterning in meiosis.. PLoS Genet.

[R5] Li Q, Saito TT, Martinez-Garcia M, Deshong AJ, Nadarajan S, Lawrence KS, Checchi PM, Colaiacovo MP, Engebrecht J (2018). The tumor suppressor BRCA1-BARD1 complex localizes to the synaptonemal complex and regulates recombination under meiotic dysfunction in Caenorhabditis elegans.. PLoS Genet.

[R6] Ward JD (2014). Rapid and precise engineering of the Caenorhabditis elegans genome with lethal mutation co-conversion and inactivation of NHEJ repair.. Genetics.

[R7] Yokoo R, Zawadzki KA, Nabeshima K, Drake M, Arur S, Villeneuve AM (2012). COSA-1 reveals robust homeostasis and separable licensing and reinforcement steps governing meiotic crossovers.. Cell.

